# Analyzing the Study of Using Acupuncture in Delivery in the Past Ten Years in China

**DOI:** 10.1155/2014/672508

**Published:** 2014-02-13

**Authors:** Yingru Chen, Xuekai Zhang, Yigong Fang, Jinsheng Yang

**Affiliations:** ^1^Institute of Acupuncture and Moxibustion of China Academy of Chinese Medical Sciences, Beijing 100700, China; ^2^Clinical and Cognitive Sciences Research Group, Faculty of Medical and Human Sciences, Institute of Brain, Behaviour and Mental Health, Salford Royal Hospital, University of Manchester, Salford M6 8HD, UK; ^3^Third Department of Neurology, Dongzhimen Hospital, Beijing University of Chinese Medicine, Beijing 100700, China

## Abstract

The use of acupuncture in inducing delivery has a long history in China. With progress over time, it has been applied in many aspects. For further study of acupuncture in delivery, this paper analyzed the papers using acupuncture in delivery in the past ten years in mainland China. 87 literatures were picked out by searching relevant electronic databases and bibliographies of relevant journals. The analysis showed randomized controlled trials that were the major type of research, while preclinical researches and literature reviews only account for around ten percent, respectively. Clinical researches indicated that acupuncture can relieve labor pain, promote maternal uterine contraction, shorten birth process, and treat postpartum disorders. Preclinical researches found that acupuncture can adjust certain hormones and improve uterus contraction of late-stage pregnant rats. However, due to lack of large multicenter randomized controlled clinical trials, standardized evaluations of clinical effects in clinical researches and detailed mechanism study in preclinical researches and unequivocal conclusions about the effectiveness, efficacy, and mechanisms of acupuncture in this field cannot be obtained from those researches yet. Further clinical and preclinical studies about the use of acupuncture in delivery with improved methodology is still needed.

## 1. Introduction

Acupuncture involves the insertion of different types of needles into the skin and subcutaneous tissues at specific points (named as acupoints) on the body, which has been used for more than two thousand years in China. Its usage in inducing delivery was recorded more than one thousand and six hundred years ago (was first recorded in Mai Jing (The Pulse Classic), wrote by Shuhe Wang in Xi Jin Dynasty (265–316 A.D.)) [1]. Acupuncture for inducing labor has been developed in a method of stimulating the onset of labor, alleviating labor pain, ripening the cervix, and even inducing intrauterine fetal death over time. For further investigation of acupuncture in delivery, and provide researchers an overview of this field in China, this paper will analyze the studies of using acupuncture for inducing delivery in the past ten years (2002~2012) in mainland China.

## 2. Search Strategy

Acupuncture and delivery/labor, acupuncture and obstetrics, acupuncture and moxibustion and labor/delivery, acupuncture and moxibustion, and obstetrics were key words, abstract, and topic list to search for relevant articles in the Chinese Journal Full—text Database (CJFD), China Science and Technology Journal Database (CSTJ) and Chinese Biomedical Literature Database (CBM) between year 2002 and 2012. Meanwhile, bibliographies of relevant journals were also manually searched. All controlled trials, experimental researches, and literature reviews involving acupuncture and delivery/labor were collected. Case reports were excluded.

## 3. Literature Overview

**Figure 1:**
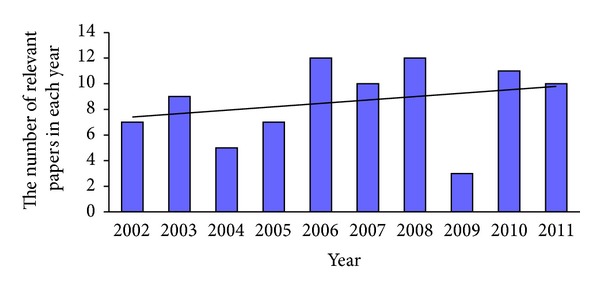
The bar chart of numerical analyses of papers regarding acupuncture inducing delivery during 2002 and 2012.

Figure 1 shows the change in numbers of relevant papers about acupuncture adopted in delivery published in each year during 2002 and 2012. It clearly indicates that the number of papers rose steadily from 7 to 12 during 2002 and 2010, though with two major fluctuates occuring in 2004 and 2009. The type and topics of those literatures will be introduced in detail as follows.

## 4. Types of Studies

Those 87 papers can be roughly categorized into clinical researches, which accounts for 77% (67/87); preclinical researches, accounting for 10.3% (9/87); and reviews 12.5% (10/87). Of 67 clinical research papers, there are 40 randomized controlled trials (RCT), four nonrandomized concurrent control trial (NRCCT), and 23 prospective case series studies (PCSS) and one questionnaire survey. There are nine ordinary reviews and one meta-analysis in those ten review articles. Detailed literature category is shown in Table 1.

Obviously, clinical researches account for more than two-thirds of those studies, while preclinical researches only account for about ten percent. Thus, the research regarding acupuncture usage in delivery in mainland China is focused on clinical research. Detailed informations are shown below.

## 5. Evaluation of Clinical Papers with Oxford Levels of Evidence

The evidence level of clinical papers (67 original papers and one meta-analysis) was evaluated by Oxford Centre for Evidence-Based Medicine Level of evidence (Oxford' level of evidence). Levels of evidence were rated from high to low as I, II, III, IV, and V. Definition of each level is shown in Table 3. Overall results showed that 60.3% literatures were Level II evidence, 4.4% were Level III evidence, 35.3% were Level IV evidence, and no literatures were Level I and V evidence. Detailed evaluation results are shown in Table 2. And Table 4 showed each articles' levels of evidence using Oxford's level of evidence.

## 6. Clinical Research

There are five major topics in those clinical researches, including acupuncture for pain relief during delivery, acupuncture for the process of labor, acupuncture for the disorders of postpartum (such as postpartum uroschesis, postpartum abdominal pain, and insufficient lactation), acupuncture for abortion, and the influence of psychological factors in the pain relieving effects of acupuncture during delivery.

### 6.1. Acupuncture for Pain Relief during Delivery

There were 22 papers published in the past decades about the use of acupuncture in relieving pain during delivery [3–22, 24, 25]. Of those papers, there were 20 reported as RCT [3–22] and 2 reported as NRCCT [24, 25]. Interventions include body acupuncture [3–11, 24], scalp acupuncture [12], acupressure [13, 25], combination of auricular acupressure and body acupuncture [14], acupoint injection [15, 16], and the combination of acupuncture or moxibustion with herbal or modern medicine [17–22]. In the 22 clinical researches, there were 14 researches that applied blank control, three that applied placebo acupuncture control, and five that used analgesic drugs control. Subjective outcomes adopted in those researches include the Visual Analogue Scale (VAS), Keele pain scores, degree of satisfaction of analgesia, and outcome of delivery. Objective outcomes include the active stage and the second birth process, the Apgar scores of new-born baby, postpartum hemorrhage amount, and adverse reactions. And plasma endorphin and 5-hydroxytryptamine concentration were measured to reveal the mechanism [6]. At last, all researchers found out that acupuncture can significantly relieve the pain during delivery, without adverse effects to both mother and child, thus concluding that acupuncture may be an economic and convenient therapy in relieving pain during delivery.

### 6.2. Acupuncture for the Process of Labor

Acupuncture was also intensively researched in induction of labor, or shortening the length of delivery [26–36]. In the 11 clinical reports, eight applied RCT design, and three applied observational research design. Of those eight RCT researches [26–30], there were five articles that used blank control and 3 that used oxytocin treatment control [32–34]. Interventions include body acupuncture [26, 28–36] (including manual acupuncture and electroacupuncture (EA)) and acupressure [27]. Outcomes include the whole duration of delivery [26–28, 35, 36], the length of second trimester of pregnancy [29, 30], the retention of placenta [31] and the uterine contraction strength [32–34]. Researchers found out that acupuncture can effectively reduce the duration of delivery (the whole length of delivery or second trimester of pregnancy), strengthen the uterine contraction, decrease the amount of oxytocin given during delivery, and significantly extend the length of uterine contraction and shorten the intermittent periods of contraction. Moreover, no adverse event occurred.

### 6.3. Acupuncture for Disorders of Postpartum Period

Researches of acupuncture for disorders of postpartum period [37–67] focused on the urine retention [37–41, 48–62] in the past ten years [37–67]. In the 31 clinical reports, there were nine researches designed as RCT [37–45], two designed as NRCCT [46, 47], and 20 designed as observational research [48–67]. In the nine RCT researches, there were three applied blank control [38, 42, 43], four applied Neostigmine treatment control [37, 39, 41, 45], and two applied herb prescription treatment control [40, 45]. Interventions include body acupuncture [37–41, 47–52, 54–63, 66] (including manual acupuncture and EA), combination of acupuncture and moxibustion [42, 44, 64], or herb [43, 45, 46, 53, 65] or Guasha [67]. Researchers found that either the single use of acupuncture or the combination of acupuncture and herbal medicine can relieve urine retention and achieve very high satisfaction. Meanwhile, studies of acupuncture in treating stress urinary incontinence [46, 47, 64], promoting recovery after cesarean section [43], treating abdominal pain [65], vaginal bleeding [44, 67], and lactation difficulty [45, 66] of postpartum had satisfactory results.

### 6.4. Acupuncture for Abortion

There were clinical practitioners using acupuncture in abortion. However, acupuncture was all used in the combination of mifepristone and misoprostol in such researches [68–70]. All three researches designed as RCT and applied misoprostol treatment control. Interventions of those researches include EA and manual acupuncture. Researchers found that EA not only had auxiliary effects with such medicines, but also could reduce side effects in abortion [68–70]. While there were different effects arising from different stimulation parameters of EA and different stimulating procedures. The procedure of EA at Hegu (LI 4) first then followed by Sanyinjiao (SP 6) can significantly alleviate abdominal pain [68, 69].

### 6.5. Psychological Factors and Acupuncture Effects

The influences of psychological factors in clinical treatment are gradually recognized by medical practitioners. Researchers investigated the influence of maternal personality type on the pain relieving effects of acupuncture in delivery [71]. 175 primiparas were grouped into stability/instability and extraversion/introversion groups according to Eysenck personality questionnaire (EPQ). It was found out that personality stability could significantly affect the parturient women's satisfaction and the effectiveness of EA, while extraversion/introversion could not. However, with combination of the two factors, they found the personality effect of EA analgesia can be arranged from higher to lower as follows: introversion stability, extraversion stability, extraversion instability, and introversion instability. The assessment of maternal personality type therefore has certain application in labor analgesia methods selection.

## 7. Preclinical Research

Preclinical researches in this field focused on the effects of acupuncture on certain hormones and uterus contraction of late-stage pregnant rats.

### 7.1. The Hormone Adjusting Effects of Acupuncture

The research of hormone adjusting with EA showed that EA could significantly influence the serum level of prostaglandin E_2_ (PGE_2_), estradiol, and progestogen in late-stage pregnant rats [72–75]. Studies found that different needling retention time had different effects on serum PGE_2_, and only the combination of needling Hegu (LI 4) 20 minutes first and then adding Sanyinjiao (SP 6) five minutes can significantly increase the contents of PGE_2_, E_2_, and P in serum and the value of E_2_/P [72]. Research about different EA waveforms was conducted and indicated that different EA parameters, especially continuous wave in Hegu (LI 4) and Sanyinjiao (SP 6) can significantly increase the contents E_2_ and the value of E_2_/P [73]. Furthermore, acupuncture following special “open-close” needling time and “host-guest” needling sequence according to Linggui Bafa (Eight methods of the intelligent turtle) could delay delivery through regulating hormones [76].

### 7.2. The Uterine Contraction Effects of Acupuncture

There were also researchers that investigated the influence of acupuncture on the uterine contraction of late-stage pregnant rats [75, 77–80]. They found that acupuncturing Hegu (LI 4) or Sanyinjiao (SP 6) separately can both increase the uterine contraction, while stimulating both acupoints at the same time achieves less effect [77]. And the combination of acupuncturing Hegu (LI 4) for 20 minutes and then adding Sanyinjiao (SP 6) for five minutes achieved the maximum effects in strengthening uterine contraction [78]. Further research to select the most effective EA parameters of this formula (acupuncturing Hegu (LI 4) for 20 minutes and then adding Sanyinjiao (SP 6) for five minutes) on uterine contraction showed that EA with sparse-dense wave (2 Hz sparse wave and 50 Hz dense wave, alternately) was more effective than other EA parameters in increasing uterine contraction amplitude, frequency, and lasting time of contraction waves of late-stage pregnant rats [79]. There was research showed that "close-host-guest needling" can reduce uterine contraction, when the acupuncture followed special protocol of Linggui Bafa [80].

## 8. Literature Reviews

The literature reviews also encompass a broad spectrum of topics in this field. There were reviews of published papers about initiating or inducing labor with acupuncture [23, 81–83], the traditional theories of avoiding acupuncture on specific points during pregnancy [84], relieving labor pain with acupuncture, or other alternative treatment [85–87]. And there were also reviews about treating urine retention with acupuncture [88].

## 9. Conclusion 

It is quite clear that the number of articles about the use of acupuncture in delivery reached its peak in 2006, 2008, and 2010, in the past ten years. It shows greater interest among clinical practitioners about using acupuncture in delivery. However, there were also fluctuations in the number of relevant papers, which shows the insufficient consistence in this field.

There was high diversity in the aims of researches in this field in the past ten years. Aims of those clinical researches about the use of acupuncture in labor had extended from relieving pain during labor and treating postpartum uroschesis to inducing abortion, shortening delivery time treating insufficient lactation, and so forth. Aims of preclinical researches also had expanded from the uterus contraction effects to hormone-adjusting effects. This just provided some clues for the use of acupuncture in obstetrics. Acupuncture actually can be used to treat many disorders before, during, and after pregnancy. This review, however, just analyzed studies of using acupuncture in delivery in the past ten years in mainland China. Papers about the use of acupuncture in treating other relevant disorders in obstetrics and gynecology can be reviewed separately in the future.

Although many studies produced encouraging results regarding different uses of acupuncture in delivery, unequivocal conclusions about its effectiveness and efficacy cannot be reached from researches in mainland China during the past ten years. Because there are many drawbacks in those studies, such as lack of large multicenter randomized controlled clinical trials, no standardized evaluations of clinical effects, and not enough detailed mechanism investigation rather than serum hormone influence. There was also research that showed that acupuncture has no influence on the rate of normal labor and neonatal asphyxia [23]. Thus, further investigations about the use of acupuncture in delivery should focus on the scientific evaluation of its clinical, biochemical, and morphological effects with large scale randomized clinical trials and well-designed animal experiments, to reveal the real advantages and disadvantages of acupuncture in obstetric.

However, without harmful teratogenic effects, acupuncture, in theory, is an ideal management for childbirth. It is simple, practical, cheap, and safe for the women and her infants. Although there were still no unequivocal conclusions of acupuncture in delivery in mainland China, with the wealth of information favoring acupuncture, it should be considered as an alternative treatment of primary health care in delivery.

## Figures and Tables

**Table 1:** The results of literatures classification according to its purpose (*n* (%)).

Purpose	RCT	NRCCT	PCSS	Review	Preclinical research	Clinical survey
Acupuncture for pain relief during delivery (*n* = 28)	20 (71.4)	2 (7.1)	0	5 (17.9)	0	1 (3.6)
Acupuncture for the process of labor (*n* = 24)	8 (34.8)	0	3 (8.7)	4 (17.4)	9 (39.1)	0
Acupuncture for disorders of postpartum period (*n* = 32)	9 (27.3)	2 (6.1)	20 (63.6)	1 (3)	0	0
Acupuncture for abortion (*n* = 3)	3 (100)	0	0	0	0	0

RCT: randomized controlled trial; NRCCT: nonrandomized concurrent control trial; PCSS: prospective case series studies.

**Table 2:** Articles' levels of evidence using Oxford's level of evidence (*n* (%)).

Literatures	Level				
	I	II	III	IV	V
Acupuncture for pain relief during delivery (*n* = 23)	0	20 (87)	1 (4.3)	2 (8.7)	0
Acupuncture for the process of labor (*n* = 11)	0	9 (81.8)	0	2 (18.2)	0
Acupuncture for disorders of postpartum period (*n* = 31)	0	9 (29)	2 (6.5)	20 (64.5)	0
Acupuncture for abortion (*n* = 3)	0	3 (100)	0	0	0

Total (*n* = 68)	0	41 (60.3)	3 (4.4)	24 (35.3)	0

**Table 3:** Oxford center for evidence-based medicine levels of evidence (March 2009).

Level	Therapy/prevention
1a	SR (with homogeneity^1^) of RCTs
1b	Individual RCT (with narrow confidence interval^2^)
1c	All of none^3^
2a	SR (with homogeneity^1^) of cohort studies
2b	Individual cohort study (including low quality RCT; e.g., <80% followup)
2c	Outcomes research; ecological studies
3a	SR (with homgeneity^1^) of case control studies
3b	Individual case control study
4	Case series (and poor quality cohort and case control studies^4^)
5	Expert opinion without explicit critical appraisal or based on physilolgy, bench research, or “first principles”

Notes: Produce by Phillips et al. [2], since November 1998. Updated by http://www.cebm.net/index.aspx?o=1025. Users can add a minus sign “−" to denote the level of that fails to provide a conclusive answer, because:

• EITHER a single result with a wide confidence interval;

• OR a Systematic Review with troublesome heterogeneity.

“Such evidence is inconclusive, and therefore can only generate Grade D recommendations”.

Grade D means: level 5 evidence or troublingly inconsistent or inconclusive studies of any level.

^1^By homogeneity we mean a systematic review that is free of worrisome variations (heterogeneity) in the directions and degrees of results between individual studies. Not all systematic reviews with statistically significant heterogeneity need be worrisome and not all worrisome heterogeneity need be statistically significant. As noted above, studies displaying worrisome heterogeneity should be tagged with a “−” at the end of their designated level. ^2^See note above for advice on how to understand, rate and use trials or other studies with wide confidence intervals. ^3^Met when all patients died before the Rx became available, but some now survive on it; or when some patients died before the Rx became available, but none now dies on it. ^4^By poor quality cohort study we mean one that failed to clearly define comparison groups and/or failed to measure exposures and outcomes in the same (preferably blinded) objective way in both exposed and nonexposed individuals and/or failed to identify or appropriately control known confounders and/or failed to carry out a sufficiently long and complete followup with patients. By poor quality case control study we mean one that failed to clearly define comparison groups and/or failed to measure exposures and outcomes in the same (preferably blinded) objective way in both cases and controls and/or failed to identify or appropriately control known confounders.

**Table 4:** Articles' levels of evidence using Oxford's level of evidence (*n* (references)).

Literatures	Level				
	I	II	III	IV	V
Acupuncture for pain relief during delivery (*n* = 23)	0	20 [3–22]	1 [23]	2 [24, 25]	0
Acupuncture for the process of labor (*n* = 11)	0	9 [26–34]	0	2 [35, 36]	0
Acupuncture for disorder of postpartum period (*n* = 31)	0	9 [37–45]	2 [46, 47]	20 [48–67]	0
Acupuncture for abortion (*n* = 3)	0	3 [68–70]	0	0	0
